# IgG Against Dengue Virus in Healthy Blood Donors, Zanzibar, Tanzania

**DOI:** 10.3201/eid2003.130150

**Published:** 2014-03

**Authors:** Francesco Vairo, Emanuele Nicastri, Salma Masauni Yussuf, Angela Cannas, Silvia Meschi, Mwanakheir AA Mahmoud, Azza H. Mohamed, Paul Mohamed Maiko, Pasquale De Nardo, Nazario Bevilacqua, Concetta Castilletti, Antonino Di Caro, Vincenzo Racalbuto, Giuseppe Ippolito

**Affiliations:** “L. Spallanzani” National Institute for Infectious Diseases, Rome, Italy (F. Vairo, E. Nicastri, A. Cannas, S. Meschi, P. De Nardo, N. Bevilacqua, C. Castilletti, A. Di Caro, G. Ippolito);; Directorate General of Development Cooperation, Rome (F. Vairo, V. Racalbuto);; Ministry of Health, Unguja, Zanzibar, Tanzania (M.A.A. Mahmoud, A.H. Mohamed, S.M. Yussuf);; Mnazi Mmoja Hospital, Unguja, Tanzania (P.M. Maiko)

**Keywords:** dengue, seroprevalence, Zanzibar, viruses, vector-borne infections, *Suggested citation for this article*: Vairo F, Nicastri E, Yussuf SM, Cannas A, Meschi S, Mahmoud MAA, et al. IgG against dengue virus in healthy blood donors, Zanzibar, Tanzania. Emerg Infect Dis [Internet]. 2014 Mar [*date cited*]. http://dx.doi.org/10.3201/eid2003.130150

## Abstract

We conducted a seroprevalence survey among 500 healthy adult donors at Zanzibar National Blood Transfusion Services. Dengue virus IgG seroprevalence was 50.6% and independently associated with age and urban residence. These data will aid in building a surveillance, preparedness, and response plan for dengue virus infections in the Zanzibar Archipelago.

In eastern Africa, the available evidence indicates that dengue virus serotypes 1, 2, and 3 (DENV-1, -2, -3) are common causes of acute fever (*1*). A recent map of DENV transmission has shown that the virus could be transmitted in most eastern African countries, including mainland Tanzania and the Zanzibar Archipelago (*2*).

In 2010, a PROMED report raised concerns about DENV infections in Tanzania (*3*). That same year, travelers from Europe and Japan were found to be infected with DENV-3 after they returned from mainland Tanzania or Zanzibar (*4*–*6*). In Tanzania, seroprevalence rates for febrile outpatients in Tosomaganga (Iringa Region) and Pemba Island (Zanzibar) in 2007 (*7*) and in Moshi (Arusha Region) in 2007–2008 (*8*) were 1.8%, 7.7%, and 10.7%, respectively. To determine DENV circulation in the Zanzibar Archipelago, we assessed the seroprevalence of DENV among adult blood donors at the Zanzibar National Blood Transfusion Services (ZNBTS).

## The Study

We conducted a cross-sectional seroprevalence survey at ZNBTS from September 20 to December 10, 2011. ZNBTS is located in Stone Town, the principal city of the Zanzibar Archipelago. Ethics approval was obtained from the Zanzibar Medical Research Ethical Committee. The sample size was calculated by using methods for proportion. The estimated prevalence was set at 50% because data were not available regarding the true prevalence of the infection in the area. Considering a population of ≈1,000,000 inhabitants and a confidence level of 95%, the sample size was set at 384 donors. Sample size was then increased to 500 donors to account for those lost to follow-up.

During the study period, all consecutive adult donors attending ZNBTS, who had been screened and selected for blood donation, were enrolled in the study. Donors were screened by serologic tests for hepatitis B virus, hepatitis C virus, HIV, and *Trepomena pallidum*; they were selected for blood donation if results of all screening tests were negative. A structured interview was conducted by using a close-ended questionnaire after the donor signed the informed consent form and before the screening. 

From each enrolled person, 10 mL of venous blood was collected. After the screening tests, the remaining serum was divided into 2 aliquots: 1 was stored at −20°C at the sample processing site for performance of the IgG ELISA at Mnazi Mmoja Hospital in Unguja, Zanzibar, and 1 was dispatched to the “L. Spallanzani” National Institute for Infectious Diseases in Rome, Italy, for testing by immunofluorescence assay (IFA) for IgG. At the end of the collection phase, samples were tested by Panbio Dengue IgG Indirect ELISA kit (Inverness Medical Innovations Australia Pty Ltd, Sinnamon Park, Queensland, Australia) according to the manufacturer’s instructions. A positive ELISA result was defined as having an index value >1.1. To compensate for the low specificity of the ELISA, we tested samples by IFA with homemade slides and a mix of uninfected and DENV-2 (New Guinea C strain)–infected Vero E6 cells. The diagnostic accuracy of the IFA has been described (*9*). 

Donors were considered positive for IgG against DENV if results of both tests were positive. Discordant results were considered negative. All districts except the urban district were considered rural areas. Univariate association between DENV IgG positivity and donor characteristics was assessed by means of odds ratios (ORs) and 95% CIs, by χ^2^ for categorical values, and Student *t*-test for continuous variables. A multiple logistic regression model using a backward procedure was used. All variables were entered in the backward selection model, and a cutoff level of p = 0.10 was used for subsequent selections. Data management and analysis were performed by using STATA version 11 (StataCorp, College Station, TX, USA).

Five hundred persons consecutively attending ZNBTS were selected for blood donation and, therefore, were eligible to be enrolled in the study. Demographic characteristics of the participants are shown in Table 1. The mean age was 32 years; 97.2% of the participants were male. Most donors had a water storage container (71.2%) and/or resting water near their home (88.0%). Bed nets were used by 59.4% of the donors, but only 40% reported insecticide spraying at home.

**Table 1:** Univariate analysis of characteristics of 500 blood donors according to IgG positivity against dengue virus, Zanzibar, 2011*

Characteristic	IgG positive, n = 253, no. (%)	IgG negative, n = 247, no (%)	Total, N = 500	OR (95% CI)	p value
Age, mean (±SD)	34 (±9)	30 (±8)	32 (±9)		<0.001
Age, for 5-y increase†				1.32 (1.19–1.47)	<0.001
Age, y					
≥36	57 (39.6)	87 (60.4)	144	1	
≤25	81 (43.8)	104 (56.2)	185	1.19 (0.76–1.85)	0.444
26–35	115 (67.3)	56 (32.7)	171	3.13 (1.97–4.98)	<0.001
Sex					
M	243 (50)	243 (50)	486	1	
F	10 (71.4)	4 (28.6)	14	2.5 (0.77–8.07)	0.126
Work					
Yes	172 (52.4)	156 (47.6)	328	1	
No	80 (47.1)	90 (52.9)	170	0.8 (0.55-1.16)	0.254
Persons in household					
1-4	80 (47.1)	90 (52.9)	170	1	
5–8	129 (53.8)	111 (46.2)	240	1.3 (0.88–1.93)	0.182
>9	44 (48.9)	46 (51.1)	90	1.07 (0.64–1.79)	0.779
Bed net use					
Yes	146 (49.2)	151 (50.8)	297	1	
No	107 (52.7	96 (47.3)	203	1.15 (0.80–1.64)	0.436
Insecticide-spraying home					
Yes	107 (53.5)	93 (46.5)	200	1	
No	146 (48.7)	154 (51.3)	300	0.82 (0.57–1.17)	0.290
Flu					
Yes	40 (48.8)	42 (51.2)	82	1	
No	213 (51)	205 (49)	418	1.09 (0.67–1.75)	0.719
Resting water‡					
Yes	223 (50.7)	217 (49.3)	440	1	
No	30 (50.8)	29 (49.2)	59	1.00 (0.58–1.73)	0.981
Water storage§					
Yes	172 (48.3)	184 (51.7)	356	1	
No	82 (56.9)	62 (43.1)	144	1.39 (0.94–2.06)	0.093
Area of living					
Rural	119 (39.4)	183 (60.6)	302	1	
Urban	134 (67.7)	64 (32.3)	198	3.18 (2.21-4.69)	<0.001

*OR, odds ratio. †Age, 5-y increase is defined as the odds ratio for every 5 years of increase. ‡Resting water is defined as any presence of water resting around the house (i.e.. ponds, puddles). §Water storage is defined as any container used to collect rain water with no mention of the size and type of container.

Of the 500 blood samples, 253 (50.6%) were positive by both tests, 77 (15.4%) were positive by ELISA and negative by IFA, and 170 (34.0%) were negative by both tests. DENV IgG prevalence was 50.6% (95% CI, 46.2–54.9). Considering IFA as the reference standard, ELISA sensitivity and specificity were 100% and 68.8%, respectively. According to univariate analysis, DENV IgG–positive donors were significantly more likely to be older (OR 1.32, 95% CI 1.19–1.47) and live in urban districts (OR 3.18 95% CI 2.21–4.69) compared with DENV IgG-negative donors (Table 1). According to multivariate analysis, older age (adjusted OR [AOR] 1.42, 95% CI 1.27–1.61) and living in an urban district (AOR 4.09, 95% CI 2.72–6.17) were independently associated with DENV IgG positivity (Table 2). Moreover, borderline evidence indicated an association of positivity with the presence of resting water near the home (AOR 1.66, 95% CI 0.99–2.76).

**Table 2:** Multivariable logistic regression model of odds of having IgG against dengue virus, Zanzibar, 2011*

Covariate†	Adjusted OR (95% CI)	p value
Age, for 5 y increase‡	1.42 (1.27–1.61)	<0.001
Water storage§		
Yes	1	
No	1.66 (0.99–2.76)	0.053
Area of living		
Rural	1	
Urban	4.09 (2.72–6.17)	<0.001

*OR, odds ratio. †Also adjusted for sex, resting water. ‡Age, for 5-y increase is defined as the OR for every 5 years of increase. §Water storage is defined as any container used to collect rain water with no mention of the size and type of container.

## Conclusions

We found high DENV IgG seroprevalence (50.6%) in adult blood donors residing in the urban district. The presence of DENV IgG is independently associated with age and urban district residence. Our results, compared with the previous low prevalence rate (7.7%) detected on Pemba island and the Zanzibar Archipelagos in 2007 (*7*), suggest an endemic pattern of transmission of DENV infection in Zanzibar, similar to the situation in other African countries (*10*). Considering the progressive reduction of laboratory-confirmed malaria cases in Zanzibar and the nonspecific influenza-like symptoms of DENV primary infections, this wide DENV circulation in Zanzibar appears to be largely underdiagnosed. Patients with primary DENV infection are likely to be mistakenly treated with antimalarial drugs on the basis of clinical symptoms, as we observed on Pemba (*11*). 

We cannot determine when the donors in this study were infected. The strong association with age could be explained by the progressively longer exposure of the older donors to the risk for infection. This association could be also explained by the successful malaria vector control initiative (use of long-lasting insecticidal nets, indoor residual spraying and biolarviciding at mosquito breeding sites, and environmental management), which could account for the lower DENV prevalence in the younger population (*12*). Of note, in our study the proportion of persons who used bed nets and those who did not keep water storage containers near home was quite low.

However, before drawing firm conclusions, a few limitations must be described. First, a high proportion of participants were male. This may have been because of the low proportion of women who donate blood, a consequence of the cultural belief that women are weaker because of blood losses during menstrual periods and pregnancies. This explanation was reported by the workers at ZNBTS and is in accordance with results from a previous study on African immigrants (*13*). Our sample population was thus less representative of the general population and could have affected the results, either by underestimating or overestimating the inferred prevalence. Second, we did not attempt to detect DENV circulating serotypes in this study; nevertheless, there is evidence of DENV-3 circulation in previous reports about imported DENV cases in Europe and Japan (*4*–*6*). Third, no antibody neutralizing assay has been performed to rule out cross-reactions with other circulating flaviviruses, but epidemiologic data from Zanzibar do not indicate outbreaks of other flavivirus infections. Our data constitute the first step toward better defining the circulation of DENV in the Archipelago and toward building up a preparedness and response plan to fight DENV infection.
